# Modelling Potential Geographical Access of the Population to Public Hospitals and Quality Health Care in Romania

**DOI:** 10.3390/ijerph17228487

**Published:** 2020-11-16

**Authors:** Liliana Dumitrache, Mariana Nae, Gabriel Simion, Ana-Maria Taloș

**Affiliations:** Faculty of Geography, University of Bucharest, 1. Blv. Nicolae Bălcescu, 010041 Bucharest, Romania; mariana.nae@geo.unibuc.ro (M.N.); gabriel.simion@geo.unibuc.ro (G.S.); ana.talos@geo.unibuc.ro (A.-M.T.)

**Keywords:** potential geographical access, health care, public hospitals, Romania

## Abstract

The geographical accessibility to hospitals relies on the configuration of the hospital network, spatial impedance and population distribution. This paper explores the potential geographic accessibility of the population to public hospitals in Romania by using the Distance Application Program Interface (API) Matrix service from Google Maps and open data sources. Based on real-time traffic navigation data, we examined the potential accessibility of hospitals through a weighted model that took into account the hospital competency level and travel time while using personal car transportation mode. Two scenarios were generated that depend on hospitals’ level of competency (I–V). When considering all categories of hospitals, access is relatively good with over 80% of the population reaching hospitals in less than 30 min. This is much lower in the case of hospitals that provide complex care, with 34% of the population travelling between 90 to 120 min to the nearest hospital classed in the first or second category of competence. The index of spatial accessibility (ISA), calculated as a function of real travel time and level of competency of the hospitals, shows spatial patterns of services access that highlight regional disparities or critical areas. The high concentration of infrastructure and specialised medical personnel in particular regions and large cities limits the access of a large part of the population to quality health services with travel time and distances exceeding optimal European level values. The results can help decision-makers to optimise the location of health services and improve health care delivery.

## 1. Introduction

The provision of adequate access to health care services continues to be central in developing policies, strategies and programs for public health. At the same time, public health policies play an important role in reducing disparities in health care access and improving health outcomes [1].

One of the strategic priorities at the WHO’s 13th General Programme of Work (GPW13), which focuses on ‘achieving universal health coverage’ reflects the importance of ensuring people’s access to appropriate health services and envisages the reduction of ‘persistent barriers to accessing health services’ ([2], p. 16). The European regional health policy framework ‘Health 2020’ (target 5) fosters the idea that the ‘provision of equitable access to effective and needed services without financial burden is a fundamental right of all citizens’ ([3], p. 116).

Access to health care services generally means ‘the timely use of personal health services to achieve the best health outcomes’ ([4], p. 4) and should be considered in relation to the need, provision and use of health services. Potential access to health care implies the potential need for health services by the whole population. It reflects the availability of health care providers and facilities in an area, while realised access to care encompasses the current utilisation of health care services by the population [5]. Accessibility does not guarantee utilisation [6] and the simple presence of a health care facility does not represent a measure of access. Thus, the volume and distribution of health care resources in an area can be considered predictors of realised access.

Access to health care is a complex construct with various dimensions and determinants that many authors have outlined and conceptualised in recent literature [7]. Geographical accessibility is considered an important dimension and refers to the physical presence or location of a service that allows it to be reached easily [8]. Penchansky and Thomas define physical accessibility, one of the five dimensions of the broader concept of access, as ‘the suitability of the location of the service in relation to the location and mobility of the patient’ ([9], p. 128).

Considered from only this perspective, the geographic location of services coupled with the transportation network and implicit distance and travel time to the nearest setting can be facilitators or barriers to the use of health care services. In the same vein, lack of services nearby or long distances between patients and health care facilities, poor road infrastructure and reduced public transportation options [10] limit potential users from seeking and obtaining care, leading to unmet health care needs and increasing the overall the cost of health care. Travel time or distance from patients’ location to health care providers in a service area are indicators of spatial impedance [9,11,12] that strongly influence health care decisions as well as the use of services [13]. In general, the distance from a service is inversely associated with utilisation, but travel time, costs and availability of reliable transport are often more important [14,15,16].

Nevertheless, the geographic location of medical services creates significant disparities in health care access, which subsequently are reflected by population health outcomes. Many regions remain underserved, while the rural population in remote areas often lack access to essential health care services [17]. The shortage of health professionals and their uneven distribution at the national level can only deepen these inequalities and have adverse effects on the quality of care [18,19]. Also, the resources of the health care delivery system are not balanced well enough to provide patient-centred care to address the complex health care demands of an ageing population [20].

With an increasing urban sprawl, population growth and ageing, it is essential to explore patterns of accessibility to health care resources at the regional and local level. Therefore, the spatial planning approach and the geographical accessibility of health care resources need to be integrated [21,22]. Inequalities in physical access to medical services are resulting from the configuration of facilities, population distribution and transport infrastructure. From this perspective, the spatial component still represents a critical determinant and should not be neglected, whether we refer to the distribution of health care services or the distribution of the population in need as recipients.

The uneven distribution of health care resources at local, regional or national levels generates inequalities in both spatial arrangement and public accessibility. The spatial arrangement of health care services is considered special and unique to each region or country, as their location respects certain spatial organisation logic and health facilities are fixed units or entities, being subject to legal or administrative regulations.

In many former socialist countries, the spatial distribution of health facilities and the allocation of health resources followed strict rules, framed in an excessively centralised and state-controlled health care system (Semashko model). This made medical services, at least theoretically, more accessible to the population. Despite the ideological objective to make health care available to all, financial, organisational or bureaucratic barriers to receiving care or quality care persisted [23]. While most hospitals in Western Europe now concentrate on acute care, most hospitals in Eastern Europe continue to provide social and health care functions [24].

In Romania, the transition from socialism to a market-based economy entailed, after 1990, reforms and reorganisation of health services, the emergence of more players in the health services market, and a more flexible regulatory environment that offered opportunities for private investors. However, the private investors targeted only attractive or profitable health care sub-sectors and specific locations, just as public investments continued to shrink. These transformations determined the concentration of medical equipment, technologies and skilled health workforce, particularly in the largest hospitals in a few cities, and are reflected by differential access of the population to higher quality health care.

Although the Romanian health care system has been subject to continuous reforms after the fall of communism in 1989, and many changes have occurred at different levels, it is currently highly centralised and dominated by the public sector, with the Ministry of Health being the central administrative authority (Law 95/2006, republished in 2015). The delivery of health care services remains characterised by the overprovision of highly specialised inpatient care and underutilisation of primary and community care [25]. The lack of general practitioners (GPs) in rural areas, the weak delivery of primary healthcare services and the failure with the hospital sector reorganisation have led to an increased rate of hospital use, population accessing hospitals for simple diagnosis or routine investigations. Hospital discharges per 1000 people were higher in Romania (213) than in the European Union (EU) average (172) in 2017 [26]. The ‘hospital’ polarises the Romanian health services [27], and access to public hospitals represents a prerequisite for receiving quality care.

An extensive hospital network, which mostly dates from the communist period (1970–1980), provides inpatient care in more than 500 units, most of them state-owned, representing around 2.5 hospitals per 100,000 inhabitants [28]. However, if hospitals are ineffectively distributed and organised, their potentially positive impact on health will be reduced or even be negative [29].

The issue of physical access in relation to the location of health care services has been the subject of much work worldwide [12,30,31,32,33,34,35]. There is extensive literature focused on the geographical accessibility of health services. Many researchers address this topic from different perspectives, orientations, and applied methods: efficiency of hospital locations [36], location pattern of physicians across communities [37] measures of geographic access using physician-to-population ratios or average distance travelled to the nearest physician [38,39]. Recent advances in methodological tools, such as Geographic Information Systems (GIS), have allowed for more accurate measurements of accessibility. According to the objectives envisaged, many studies have focused on the validity of measuring geographic accessibility by estimating travel time [40,41,42,43,44], on assessing the spatial and nonspatial factors of health care access [45], differences in access causality [46], accessibility measurements errors [47], population accessibility to primary care [48] or modelling accessibility to hospitals [49]. The complexity of assessment methods has increased while studies have become more sophisticated. However, these methods need to be integrated into one framework to reveal the connections between them and compare their advantages and weaknesses [50].

More recently, researchers use web-based mapping services, like Google Maps Application Programming Interface (API), to measure the overall accessibility at multiple scales, saving significant time for data collection and complex calculations [51]. Scholars have developed tools to accurately estimate the travel time between origins and destinations, highlighting the many advantages in using API compared to the ArcGIS Network Analyst approach [52].

Despite the relevance of this topic, few studies addressed the issue of geographical accessibility to health care facilities in Romania, particularly due to the lack of input layers and limited data reliability, resulting from a highly fragmented data collection system [25,53,54,55,56].

The current study explored the potential geographical access to different types of public hospitals in Romania, by using Google Maps API. This is the first attempt to assess accessibility to hospitals at the national level, given the numerous limits imposed by the lack of data on the transport network and health care activity.

The paper is structured as follow: the first section looks over the concept of access to health care and the role of geographical location, presenting the general context and the importance of hospital access in Romania; section two presents data sources and how the model was developed and used to estimate distance and travel time from many origins (residential locations) to many destinations (hospitals) and the limitations of the study; section three presents two different scenarios of geographic accessibility highlighting spatial inequalities, discussing the disparities in access of the population to public hospitals and the implications at territorial level. A summary concludes these sections and envisages further studies.

## 2. Materials and Methods

### 2.1. Study Area

Our analysis explored the potential geographical access of the population to public hospitals in Romania. The national level serves as an outstanding study area to conduct a travel time analysis because of its geographical disparity regarding geophysical conditions (mountains 31%, hills and plateaus 36%, plains and meadows 33%) and population distribution. The total population is continuously declining from 22,810,035 inhabitants in 1992 to 19,530,631 inhabitants in 2018, due to the low birth rate and significant emigration. A large share of the Romanian population (46.3%) is still living in rural areas and the population over 60 years old surpasses 25% of the total population (5,018,620 inhabitants out of which 16.9% are over 80 years old). Both rurality and ageing population represent significant concerns, raising issues not only in terms of increasing demand for medical services but also in terms of access to them. To this is added a poor road infrastructure, summing up 86,234 km out of which only 33,928 km are modernised. The density of public roads per 100 km^2^ of territory in 2018 was 36.1. However, of total public roads, only 17,740 km are classified as national roads, while 68,494 km are county or communal ones, most of them with circulation difficulties, traffic problems and speed limits [28]. Therefore, accessibility to health care services and facilities, specifically to public hospitals, is a public health concern.

### 2.2. Methods

Measuring spatial accessibility to public hospitals is an essential step in assessing public health policy decisions. This is challenging in the case of Romania where the well-established methods reported in the literature are difficult to apply due to the lack of data accuracy and limited data source referring to the road networks: road geographical distribution and travel speed by road segments. Although extensive data on the functioning of the health system and population health status were collected, the information systems currently suffer from a high degree of data fragmentation and duplication, and the use of existing data in planning and decision-making is poor [27].

Starting from the spatial distribution of hospitals and their coordinates, we calculated the theoretical catchment areas [57,58,59]. Further, we are interested in quantifying the proportion of the population who utilise the nearest health care facility (the hospitals). In this case, we applied the Thiessen polygons method [60] that provides a reasonable approximation of patient behaviour and assumes a uniform utilisation rate through that population. Polygons generated from each hospital point were used to define the hospital catchment area so that any settlement inside the polygon is closer to that hospital than any of the other hospitals.

The next step consisted of the calculation of other significant parameters of potential geographical accessibility such as the travel time and distance by car from the settlement’s centroid to the nearest hospital, using Distance API Matrix from Google. Although less advanced from a conceptual point of view, travel time or distance to the nearest hospital might be more meaningful, intuitive and transparent for health professionals, policymakers and planners [61]. The use of time and the interrelationship between space and time has an important effect on choice. Therefore, travel time is more important than travel distance [62].

### 2.3. Data

#### 2.3.1. Population Data

This study used Local Administrative Units (LAU2) consisting of municipalities or equivalent units as the basic evaluation units of travel time to the nearest hospital. The populations of the LAU2 range from 133 to 1,916,017 inhabitants.

The National Institute of Statistics provides public population datasets at the level of the settlement, which means that the resident’s home location is unavailable. Given this situation, all of Romania’s settlements were geocoded at the centroid level. The centroid was considered as representing the total population of the corresponding settlement. The settlements layer covering 13,277 territorial units was considered for geocoding and travel time and distance computing process.

#### 2.3.2. Hospitals and Physicians Data

Data referring to hospitals and physicians for the period 2012–2019 were available at the National Institute of Statistics database (NIS), Diagnosis Related Groups system (DRG) managed by National School of Public Health, Management and Professional Development (NSPHMPD), Romanian Ministry of Health database and the actualised websites of each hospital.

The selection of hospitals for which accessibility was estimated, has taken into account the types of hospital, as they are defined in Romania by Hospital Law no 270/2003, Law no 95/2006 and Order of Ministry of Health no 323/2011. The main criteria considered were their territorial location, the complexity of services and their level of competency. Thus, from the territorial location point of view, regional, county, municipal and town hospitals have been selected. During the hospital selection process, we considered the specific pathology for which they can provide services, choosing only the general hospitals and the emergency hospitals that can offer multiple services for various diseases. Specialist hospitals, assisting a single pathology or chronic diseases hospitals were eliminated.

The postal addresses of hospitals, classified by categories, were obtained from the Romanian Ministry of Health. Then each hospital was automatically geolocalised (latitude, longitude, World Geodetic System-WGS 84 Web Mercator) through Google Maps. Since in several cases, the postal addresses were not recognised by Google Maps, we manually localised and refined the geographical position using as base layer the map or satellite images. One of the data preparation requests is that both the origin and destination layers need to use the same geographic projection. All the GIS layers of this research were (re) projected to WGS 84. In total, 366 hospitals were incorporated within a Geographic Information System by geocoding their addresses. Since not all hospitals have general addressability, we took into consideration a list of 228 hospitals according to the criteria mentioned above.

To observe the population accessibility to hospitals, two scenarios at two different spatial levels were generated, depending on the categories of competency of hospitals (I, the highest to V, the lowest, according to the Ministry of Health). The first level displays accessibility to all 228 selected hospitals, while the second level presents the population access to the hospitals classified in the first and second categories.

#### 2.3.3. Google Maps API Data

The distance and travel time on the road between hospital locations and patients are key elements in the process of modelling the accessibility of the population. Travel time is the most common indicator for measuring access through traditional methods of origin-destination (O–D) matrices in GIS analysis. However, the navigation and web-mapping platforms provide more substantial and accurate geographical data [51,63]. Online sources such as Google Maps, Here We Go, and Bing Maps are useful tools in the evaluation of the shortest routes in terms of time and distance for the public [64].

The challenge for researchers is finding a way to automate the process of obtaining a travel time matrix from many origins to many destinations. In this context, we selected Google Maps Application Programming Interface (API), a service that provides travel distance and time from an origin point (O) to a destination (D) (O–D travel time matrix). Google maps routing algorithm is a reliable source to estimate O–D travel time with minimal data preparation and GIS software knowledge [52]. The information returned is based on the recommended route as calculated by Google Maps API. For each centroid settlement, the best car route recommended by Google is considered its accessibility to the hospital. As traffic conditions have a strong influence on travel time by car, the data were acquired at the non-peak travel period from 9:00 to 11:00 p.m. in order to avoid both the traffic congestion of working hours and the low traffic intervals during the night.

### 2.4. Accessibility Indicators

GIS datasets for modelling health care accessibility presume access to different digital layers on land-use, population, health care units, and roads. Ideally, the information must have spatial resolution and sufficient accuracy in separating similar patterns as well as access to official statistical data to be transferred into GIS format.

The best route recommended and the minimum travel time generated serve as appropriate indicators for measuring spatial accessibility. Based on the minimum travel time from each settlement to the nearest hospital, the study used Inverse Distance Weighting (IDW) interpolation to generate national-level maps of accessibility to different hospital categories. Furthermore, the travelling time was summarised at LAU2, and the accessibility to all categories of the hospitals was divided into several levels using a time scale: within 30 min, 30–90 min, 90–120 min and over 120 min. The same scale was used to map population accessibility to hospitals belonging to category I and II, considered together.

#### Accessibility Index (ISA)

In order to quantify the population’s accessibility to public hospitals, an Index of Spatial Accessibility (ISA) was calculated. It quantifies spatial accessibility as a function of travel time from origin O (settlement centroid) to destination D (the nearest hospital), taking its category into account.

The following formula was used:

(1) $${\mathrm{ISA}}_{settlement}=H_{r}\frac{1}{T_{m}}$$

where $H_{r}$—Hospital rank, from I to V, according to the Ministry of Health, weighted with values from 5 to 1, 5 for rank I hospital and 1 for rank V hospitals; $T_{m}$—Travel time (minutes) from settlement’s centroid to nearest hospital, *n*—number of settlements in LAU2 and LAU2—local administrative units (villages, towns and municipalities). The ISA facilitated the analysis of spatial patterns of access to hospitals for each administrative unit (LAU2), highlighting travel time and the hospital category to which localities in LAU2 have access. This indicator summarises the time required to access the nearest hospital and the type of services provided there, as asserted by the categories of competency to which the hospital belongs:

(2) $${ISA}_{LAU2}=\frac{1}{n}\overset{n}{\underset{i=1}{\sum }}{ISA}_{settlement\_i}$$

In light of the lack of reliable statistical data to create weights for hospital competency, we arbitrarily assigned weights, using simple and intuitive values to allocate more influence to hospitals that have higher competency, according to the 2011 Ministry of Health classification.

The ISA incorporates two effects. It increases with the competency of the hospital and if the time to reach the hospital decreases. Index values close to 1 mean good access, suggesting reduced travel time to hospitals that can provide complex services, usually belonging to the first or second category. Conversely, values close to 0 show limited or poor access, and imply longer travel time to the nearest hospital, which belongs to the low-competency category. Mapping these spatial patterns may be used to identify regional disparities or to highlight critical areas where more resources can be allocated to the development of both medical and transport infrastructure.

### 2.5. Study Limits

This exploratory study nevertheless has limitations. The authors had to adapt their model and methods to the available data. The lack of data regarding the transport network and the low reliability of the data concerning the hospitals’ activities made it difficult to apply already validated methods that can shed light on the situation more accurately.

The dimensions of the study area can influence the accuracy of the results. Conducting a study at the national level, with such a variety of geographical conditions and limited data is challenging and could generate several errors. Also, using an origin point that is less accurate than the patient’s home address has the potential to reduce the accuracy of the results, as it may influence the route taken, affecting the distances and travel times [65].

Because the volume of Origin-Destination (O-D) queries is restricted to 2000 per day, so we limited our queries (over 13,000) to a single time slot, where road traffic is reduced all over the country. Travel times based on road networks and speed limits are sensitive to traffic congestion. Road congestion circumstances could significantly affect travel time to a hospital or even the distance to a medical unit, if alternative routes are suggested to avoid congested areas, making access more difficult. We plan to test different time intervals, considering both peak and non-peak hours, thus obtaining a more accurate model in forthcoming studies.

In addition, the arbitrary nature of weights assigned to hospital competency can also affect the results. While the spatial distribution of ISA values highlight and confirm the best situations for people living in large cities and metropolitan areas and the worst situations for people living in rural remote areas, a more detailed analysis is needed for ISA values that reflect medium level access.

## 3. Results and Discussion

### 3.1. The Hospital: A Central Role in the Delivery of Health Care in Romania

Hospitals are essential components of health systems and still play a central role in the delivery of healthcare in Romania. Despite numerous reforms to reduce hospital capacity and strengthen primary care, the ‘hospital’ has proven to be a complex institution, resistant to change, which continues to dominate the landscape of care in Romania.

Based on territorial location, there are regional hospitals, county hospitals and local hospitals (municipal, town or communal). The specific pathology was used to differentiate hospital by types: general hospitals, with a minimum of two primary specialities; emergency hospitals, with a complex structure of specialities, medical equipment and appropriate specialised personnel; specialist hospitals that provide medical care in one particular speciality; and chronic diseases hospitals, ensuring long-term care for different chronic diseases. From an education and research point of view, there are clinical hospitals, with university departments and institutes, usually organised in university centres (Law 95/2006) [66].

In terms of ownership, hospitals can be public, public with private sections or private. However, inadequacies in the criteria led to confusion when some county hospitals doubled as regional, emergency or clinical hospitals. Local hospitals could be general or emergency hospitals, public or private or both.

**Figure 1:**
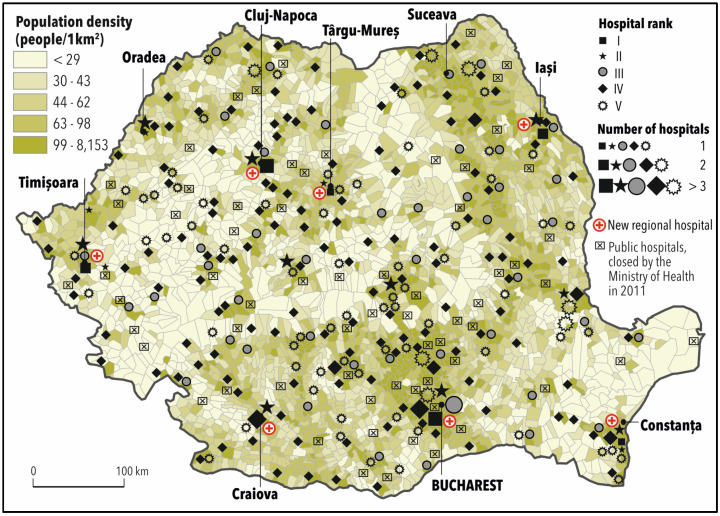
Distribution of hospitals according to the level of competency (2011).

Recently, Ministry of Health Order no 323/2011 classified Romanian hospitals according to their level of competency in five categories, from very high or high (categories I and II) to low (categories IV or V) [67]. Various criteria were considered: number of specialities, level of complexity of services provided, equipment, and specialised medical personnel, according to which most Romanian hospitals belong to category IV or V, having limited competency and being unable to provide complex medical services. The territorial distribution of high or medium competency hospitals in Romania is consistent with those of the large cities like Bucharest (19), Cluj-Napoca (4), Iași (2), Timișoara (2), Târgu-Mureș (1) and Constanța (1) (Figure 1).

The national reporting system summarises health care providers in the public and private system as follows: 567 Hospitals, 146 Polyclinics, 11,252 GP offices, 10,754 Health specialised offices 14,879 Dentist offices, and 9495 Pharmacies [28]. In terms of ownership, most of the hospitals (366) are public while most of the GPs, dentist offices and pharmacies are private.

The number of hospitals increased after 2011, from 464 units to 567 in 2016, as well as the number of hospital beds, from 6.4 to 6.7 beds per 1000 inhabitants. This was mostly due to the private health sector. So also, 67 public hospitals were closed in 2011, but most of them were reopened immediately after the closure decision or in subsequent years, following population protests or local authorities’ request.

Even if the private hospital sector is growing in size and complexity, it remains very inaccessible to the majority of the population due to its high costs and its being in the large cities. They are equipped with modern medical devices and tend to have a more friendly doctor-patient relationship. However, most of them have a limited number of specialities and cannot treat severe emergency health conditions.

After 1990, there were many attempts in Romania to adopt organisational models that had proven successful in other Western European countries. However, specific structural deficiencies, as well as local factors associated with the political, social and cultural environment, have undermined these efforts to reform the health care system (HCS).

The underfunding of the HCS significantly affected the fulfilment of some reform measures over time. More recently, health workforce shortage and the unequal territorial distribution of health professionals make the delivery of health care and access of the population to quality medical services difficult.

The shortage of health professionals is one of the main ongoing challenges the health sector is facing. This is mainly due to significant emigration of physicians, after Romania’s EU accession (2007). The health workforce, according to NIS (2018), relies on 60,585 physicians, out of which over 60% are practising in the public sector, and 145,317 nurses, meaning 322 inhabitants per physician and 134 inhabitants per nurse, which is below the European average (Table 1). While the number of specialist doctors is increasing, from 32,568 in 2010 to 42,309 in 2017, the number of general practitioners decreased in the same period from 14,509 to 12,184 [68]. The geographic distribution of the health workforce is uneven across the country with rural areas being disadvantaged.

Large urban centres have the highest number of doctors in the public and private sectors, with 66% of the medical workforce concentrated in 6 large cities. Shortages of physicians and other health care professionals are more acute in small towns or rural areas, where 5% of the localities have no doctors [69].

These deficiencies explain why a large number of cases admitted to hospitals (75%) do not have a referral from the family doctor, and 50% of patients are seeking care in the emergency room for issues that would be better addressed in the primary care settings [70].

Therefore, in Romania, avoidable hospitalisations represent 7% of all hospitalisations, this high rate reflecting reduced accessibility or availability of basic health services or the under-utilisation of primary care [71].

### 3.2. Modelling Potential Geographical Access of the Population to Public Hospitals

#### 3.2.1. Potential Geographical Access of the Population to All Categories of Hospitals

**Figure 2:**
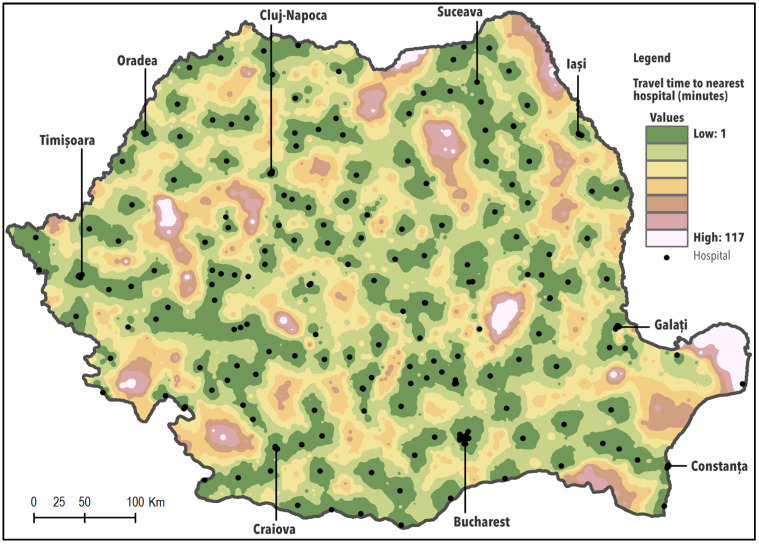
Access to hospitals (all categories).

To quantify variation in population accessibility to hospitals, we considered the shortest routes from the villages to the nearest hospitals. We relied on the web path planning API to obtain traffic navigation times between two points and we chose the shortest time path. We assumed that the population can travel by personal car. We categorised the access time by roads to the nearest hospital into four classes, ranging from less than 30 min, 30–90 min, 90–120 min, and over 120 min. Figure 2 shows how geographical accessibility, calculated by using IDW Interpolation, varies across Romania.

When considering all categories of hospitals, the potential physical access of the population to hospital care appears relatively good, as over 80% of the Romanian population can reach hospital facilities in less than 30 min of driving. In contrast, the maximum travel time to the nearest hospital by car is 117 min and 0.1% of the population is unable to obtain care, not having road access.

Geographical distribution of the most accessible areas overlays the urban spaces and highly populated territories surrounding large cities such as Bucharest, the capital of the country or Iași, Timișoara, Cluj-Napoca, Craiova, with an important role at the regional level and a relatively good hospital and road infrastructure. Areas with reduced accessibility are usually located in mountainous regions or Danube Delta and are generally sparsely populated (Figure 2).

The summary of the population’s geographical accessibility is provided in Table 2. Based on these data, 17% of the Romanian population live within 30–90 minutes’ drive time to a hospital while those requiring a longer drive time to the closest facility represent less than 0.2% (Table 2).

Despite this situation, people often face significant barriers to receiving needed and timely care. Many municipal hospitals are poorly equipped and are suffering a dramatic shortage of specialised health professionals and are unable to provide complex medical services. They are delivering only basic services and refer more complex cases to hospitals with a medium or higher level of competency (I or II). Some population groups have reduced capacity to travel due to age, costs, or lack of transportation. Most of them live in small towns, or remote rural areas and are unable to obtain care due in part to the long distance.

The lack of health care resources, both equipment and specialists, in some geographic areas and the concentration of medical services in others have led inevitably to significant variations in hospital utilisation rates, with large disparities being observed in regions and cities within the country. Low rates might reflect the inefficient use of resources and unmet needs, whereas high rates depict over-provision of resources and unnecessary interventions [72].

We analysed some indicators related to hospital activity using the Case Mix Index (CMI), calculated as a report between the total number of weighted cases and the total number of solved cases, which varied in 2019 from 0.3 to 1.9. From a total of 406 hospitals, 257 hospitals (63%) registered values under 1.0, reflecting their low capacity for solving complex cases [73]. The total number of hospital discharges (in-patients for all causes per 100,000 inhabitants) slightly declined from 23,287 in 2010 to 20,409 in 2016 [74].

However, less than 50 hospitals, mostly located in the large cities and university centres such as Bucharest, Cluj-Napoca, Iași, Timișoara, Craiova, Târgu-Mureș, Galați, Constanța, recorded 80% of the total discharges, while about 130 municipal and town hospitals registered a reduced number of cases annually.

The number of hospital beds supported by the National Health Insurance House is subject to approval by public authorities through ‘Planul National de Paturi’ (National Bed Planning) [75]. The Plan mentioned above is proposed by the Ministry of Health and approved by the government every three years. Trends indicate that the overall number of public hospital beds in Romania is decreasing from 136,341 in 2010, to just about 119,579 in 2017. This decline is in line with the reform measures aimed at reducing hospital care and increasing the efficiency of medical services.

Although the number of hospital beds decreased, the occupancy rates varied across regions and hospital sizes. Many municipal or small-town hospitals are serving low-density areas and registered low bed occupancy rates while in county emergency hospitals or units belonging to higher categories of competency, the number of admissions doubled as well as bed occupancy rates. To compensate for the high number of hospital admissions and to fit into the government-approved budget, they reduce the average length of stay for in-patients. However, early hospital discharge may not lead to overall cost-savings if it results in the need for more intense subsequent health care utilization [76,77].

This contributes to the overcrowding in hospitals, overworked physicians, and the depreciation of services delivered within the hospitals concerned. At the same time, small hospitals are subject to closure or reorganisation, mostly for economic reasons. Hospital closures have a significant impact on their surrounding communities and have been associated with bad health care for the population, especially the most vulnerable residents [78].

#### 3.2.2. Potential Geographical access of the Population to Hospitals with a High Competency Level

**Figure 3:**
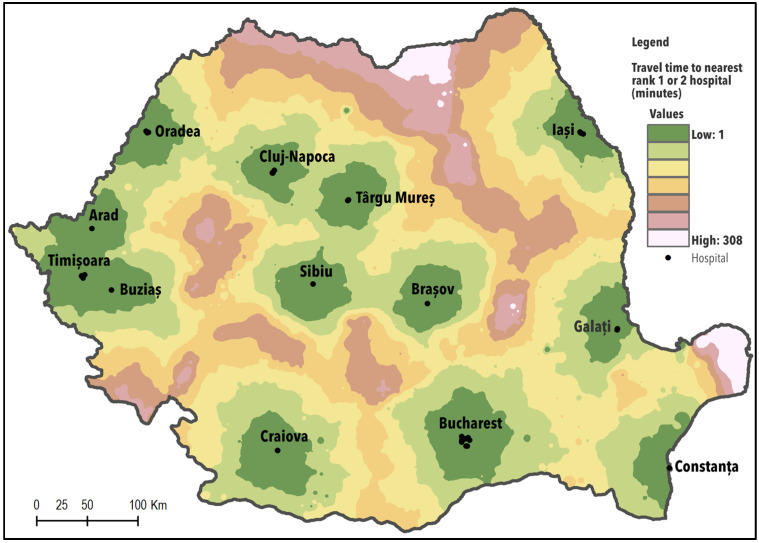
Potential geographical access to high-level competency hospitals (category I and II).

During the analysis, we focused on the population’s access to the nearest hospital belonging to categories I or II. In this context, only 29.2% of the Romanian population has to travel less than 30 min to the nearest hospital, while for 36.5% the driving time ranged between 30 and 90 min (Table 2). Geographical distribution of the areas with reduced accessibility is associated with the Carpathian region; mountain chains constitute physical barriers impacting the road infrastructure and circulation as well. Remoteness affects both rural and urban areas, and the population living in mountainous areas must travel extra time to reach the closest high competency hospital (Figure 3).

More than 3.5 million people need to travel longer than 120 min to the nearest hospital of the first and second categories and about 10,000 residents living in the areas most remote from hospitals, the Danube Delta, have no direct access, by roads, to a medical facility. Major cities in Romania polarise the neighbouring areas and the portion of the population that lives here has better access to hospitals belonging to these categories.

Access of the population to high-level competency hospitals is difficult, with travelling time that exceeds the values considered optimal at the European level. Access difficulties rely on the location of health facilities, the patterns of settlements, the population it serves and their capacity to travel for health services. Thus, people living in small towns or isolated rural areas have to travel or drive, for about 308 min, to reach the closest hospital able to deliver multiple services. This perhaps explains the higher share of the population (4.9%) in Romania with self-declared unmet needs for health care services due to either ‘financial barriers, waiting times or travelling distances’, compared to the EU average (1.8%) [79,80]. The share of the population reporting unmet needs for a medical examination because ‘it was too far to travel’ in EU28 (2018) was around 0.1%; Romania, with 0.5%, was in the third position after Estonia and Croatia.

#### 3.2.3. Index of Spatial Accessibility

**Figure 4:**
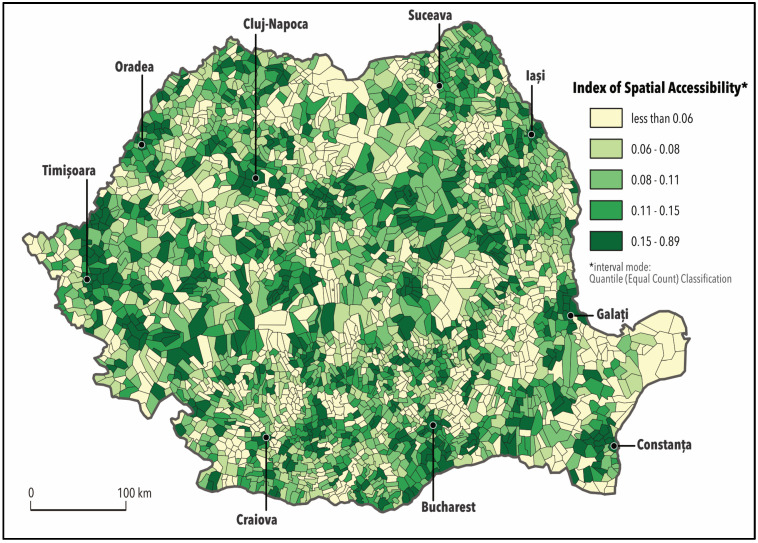
Index of Spatial Accessibility (ISA).

The Index of Spatial Accessibility (ISA) measures potential geographical access and provides an overview of hospitals’ accessibility, considering both drive time and the complexity of medical services. The values of the ISA range from 0.01 to 0.89, a value close to 1 meaning a high level of accessibility (Figure 4).

The values of the accessibility index show that 18.6% of the Romanian population, 3,940,118 people, live in areas with reduced access to hospital services (index values lower than 0.06). Nevertheless, a significant part of the population (34.6%) experiences a low and medium level of accessibility (0.06–0.11), and 14.8% benefit from relatively high accessibility. 32% of the population, which is the equivalent of 6,797,723 people, who live in large cities or metropolitan areas have high accessibility to hospital services, according to the values of the index of spatial accessibility (0.15–0.89) (Table 3).

Figure 4 presents spatial patterns of hospital accessibility. Local administrative units (LAU2) are classified into five using the quantile classification method. Since the hospitals are spatially dispersed and the travel patterns are divergent depending on the competence level and the medical services provided by the hospitals, the ISA values vary across the country, shifting inequalities among metropolitan areas, large cities, and rural areas.

The lowest ISA values highlight areas that have limited access to medical services, which translates to longer travel time and access to low-ranking hospitals, which offer only basic medical services. The regions with lower accessibility are represented with shades of light green and overlap mountain areas. The highest values, represented on the map with shades of dark green, are recorded in first-tier urban areas that usually hold hospitals of categories I and II. The population living in these areas benefit from rapid access to hospitals with a higher level of competency, multiple medical services and short travel time between their residence and hospital. Critical areas are those located in remote villages far away from urban centres or those with limited road infrastructure, where ISA presents the lowest values (less than 0.06).

Variation in geographical access to hospitals reflects the variation of distance to health care settings, which directly impacts on the use of services and can be associated with a range of poor health outcomes. It also can reflect patient behaviour or preference, or the level of hospital technology at a hospital facility [81].

Peoples living in large cities or their surrounding rural neighbourhoods with a high accessibility level have many choices and more chances to receive quality care when they need and within a relatively short driving time. Rural residents from remote areas without a car find it difficult to reach major hospitals and have to face higher costs, not only for travelling but also for the time involved; these having a disproportionate effect on the poor [82].

Strengthening primary health care, reorganising the hospital sector by reducing the number of hospital beds, and the introduction of the Diagnosis-related Groups (DRG) mechanism to reimburse hospital activities or legalising private practice were the main achievements of the Romanian Health Care system reforms. However, less attention was paid to the spatial configuration of the hospital network, and potential barriers associated with physical access to medical services were ignored. The actual configuration of the hospital network in Romania, which was inherited from the communist period, finds it difficult to meet the present needs of the community. Despite a uniform distribution at the national level, there is a large variety of hospital types across geographical regions, and their locations and services provided do not necessarily reflect the population’s needs and many people often lack access to basic health care services or do not receive timely and appropriate care.

Despite a strong legal and political commitment to improving health care efficiency, the reconfiguration of the hospital network has been challenging, due to the shortage of specialised medical personnel and the scarcity of financial resources. Maintaining a large number of hospitals, with a low level of competence, which provides only basic services to patients, are poorly staffed and partially used, is not cost-effective. At the same time, proposals to centralise hospital services often face public opposition, arising from concerns about future access to care.

The 2014–2020 Health Strategy envisages restructuring the regional hospital network to reduce the number of hospital facilities and provide integrated services to improve the coordination of treatments. Eight regional master plans on health care, one for each of the eight regions of Romania were approved by Order no. 1376/2016 [83].

The plans include the reconfiguration of hospital network through the construction of seven new hospitals in major cities such as Cluj-Napoca, Târgu-Mureș, Timișoara, Iași, Craiova, Constanța, out of which three are planned to be built in the first stage, in the South West, North-West and North-East regions of Romania.

These new regional hospitals will replace emergency county hospitals and will gather the infrastructure, workforce and expertise from the region to provide highly complex care. This will involve the redeployment of specialists across regions, practising in county or emergency hospitals to these new regional hospitals, which will increase the already existing imbalances with hospitals with low competencies remaining without specialist physicians. More than that, the locations of these new hospitals must take into consideration the travel time between facilities and transfer capacity between hospitals, road infrastructure, and ambulance response time across rural and urban areas [56]. The regionalisation of hospital services has economic and functional advantages but does not necessarily bring services closer to users.

Maintaining equitable physical access, however, implies the consideration of factors that lie beyond the health care system: spatial arrangement, road infrastructure and amelioration of transport difficulties/capacities. Geographical access models have enormous potential for informing policy development and grounding debate on how to achieve social equity of hospital access [62]. The travel time results provide opportunities for future research. The relationship between hospital accessibility and utilisation can be explored to examine the extent to which the time travelled is an expression of regional under- or over-supply rather than an expression of patient preferences.

## 4. Conclusions

This study combined the estimation of potential population flow towards hospitals and the proximity of health care availability. It aimed to use the mapping capabilities of GIS to analyse the areas with low to high accessibility to public hospitals. Web navigation services such as Google Maps API are suitable for providing real-time data of the shortest route in terms of distance and travel time from patients’ residences to the nearest hospital. The data were acquired for a non-peak travel period at 9:00–11:00 p.m., to avoid both the traffic congestion during working hours and the low traffic intervals during the night. Within this study, we considered the potential accessibility of hospitals using weights for different hospital competence level and developed an index of spatial accessibility (ISA). ISA was calculated as a function of real travel time and level of competency of the hospital. It enabled the mapping of access patterns, highlighting spatial inequalities or critical areas.

The results showed that travel time and distances to the nearest hospital services exceed the optimal values at the EU level, due to the clusterisation of health care infrastructure and highly specialised medical personnel in metropolitan regions and large cities. The spatial patterns of accessibility vary widely, with lower-ranked hospitals located in remote rural areas, towns and small cities or areas where the geophysical features of the country represent natural barriers. In contrast, higher-ranked hospitals are more accessible in metropolitan areas. Almost 4 million inhabitants live in areas with a very low level of accessibility to a hospital (experiencing longer travel time and access to lower-ranked hospitals) while 6.7 million inhabitants live in areas with a high level of accessibility to hospitals (experiencing shorter travel time and access to higher-ranked hospitals).

This approach may contribute to a better understanding of the territorial inequalities that relate to physical access to hospitals, thereby aiding the detection of clusters and spatial polarisation trends. The findings can help policymakers when developing integrated spatial planning strategies to optimise investments in health care infrastructure and reduce disparities in the geographical access of the population to medical services. Future research is needed to improve accessibility models in four areas: a more dynamic approach travel times that take into account how road congestion affects travel times during a 24-h period; more sensitive measures of population demand for hospital services; and more sensitive measures of hospital capacity and competency. Further in-depth studies need to be developed at the intra-regional and local scale where health care resources can be coordinated across administrative districts.

## Figures and Tables

**Table 1:** Health workforce structure in Romania (2018).

Medical Personnel	Total	Urban Areas	Rural Areas	Romania Average Inhabitants/Medical Personnel	EU Average Inhabitants/ Medical Personnel
Doctors	60,585	54,870	5715	322.4	262.4
out of which: GPs	12,027	7584	4443	1623.9	1088.0
Dentists	16,457	14,425	2032	1186.8	343.2
Pharmacists	17,611	14,199	3412	1109.0	1115.2
Nurses	145,317	129,424	15,893	134.4	135.6

Source: National Institute of Statistics and Eurostat.

**Table 2:** Population access to hospitals.

	All Hospital Categories		Rank I and II Hospitals	
	Population	% Total Population	Population	% Total Population
Less than 30 min	17,538,668	82.7	6,183,014	29.2
30–90 min	3,611,485	17.0	7,738,097	36.5
90–120 min	42,265	0.2	3,742,540	17.7
Over 120 min			3,528,767	16.6
No roads access	10,728	0.1	10,728	0.1

**Table 3:** Population share by levels of hospital access.

ISA Values	Accessibility Level	Population (Millions)	%
Less than 0.06	Very low	3,940,118	18.6
0.06–0.08	Low	2,884,692.0	13.6
0.08–0.11	Medium	4,444,386	21.0
0.11–0.15	Relatively high	3,136,227	14.8
0.15–0.89	High	6,797,723	32.1
Total population		21,203,146	100.0
